# The heating rate matters! contact heat evoked potentials in musicians and non-musicians

**DOI:** 10.3389/fpain.2025.1555034

**Published:** 2025-06-09

**Authors:** Fabian Sternkopf, Paulina S. Scheuren, Catherine R. Jutzeler, André Lee

**Affiliations:** ^1^ Institute for Music Physiology and Musicians’ Medicine, Hanover University of Music, Drama and Media, Hannover, Germany; ^2^ International Collaboration on Repair Discoveries, University of British Columbia, Vancouver, BC, Canada; ^3^ Department of Anesthesiology, Pharmacology, and Therapeutics, Faculty of Medicine, University of British Columbia, Vancouver, BC, Canada; ^4^ Department of Health Sciences and Technology (D-HEST), ETH Zurich, Zurich, Switzerland; ^5^ Swiss Institute of Bioinformatics (SIB), Lausanne, Switzerland; ^6^ Department of Neurology, TUM Klinikum rechts der Isar, Technische Universität München, Munich, Germany

**Keywords:** pain, CHEPS, music, nociception, neuroplasticity, heating rate, EEG

## Abstract

Classical musical training requires extreme levels of fine motor control, resulting in adaptive neuroplastic alterations in professional musicians. Additionally, musicians have a high prevalence of pain syndromes, which makes them an interesting group to research the influence of neuroplasticity on nociception. This report consists of two parts. Firstly, we present the results of a preliminary study comparing musicians and non-musicians with respect to their cortical responses to noxious heat stimuli at their hands and feet, using contact heat evoked potentials (CHEPs). Secondly, we quantitatively discuss the influence of the heating rates of two different stimulation devices on CHEPs when applying the exact same settings. For this, we measured the temperature curves of the devices’ stimuli and connected their respective heating rates to the resulting CHEPs. Musicians showed a significantly larger $N_{2}$ latency difference between hands and feet (20.86 ms, p=0.0045), compared to non-musicians. Additionally, we found that, despite the exact same settings, different stimulation devices produced considerably different temperature curves. The resulting time difference between the stimulation devices of 104.78 ms explains the latency difference of the CHEPs produced by the respective device of 104.09 ms extremely well. This study underlines that musicians are an interesting model for neuroplasticity regarding nociception, as they respond differently to nociceptive stimuli. Moreover, it contributes to the understanding of the connection between a stimulation device’s heating rate and the resulting CHEPs, an important finding that has never been quantified before but has considerable consequences on the comparability of results.

## 1 Introduction

According to a 2012 survey with 3,011 participants of the general population in Germany, 26.8% of the interviewees reported being affected by either acute or chronic pain (1). Compared to this population, professional classical musicians have a vastly higher prevalence of pain syndromes of up to 89.5% (2–4). In animal models somatosensory integration has been shown to interlink with nociception, for example by facilitating the nociceptive response via multimodal somatosensory integration (5) or by an activity dependent myelination of the nerve fibres (6–8). Specifically, the intense training of extremely refined somatosensory integration that is professional musical practice has been shown to induce structural and functional neuroplastic alterations. For instance, musicians have been shown to have an enhanced representation of their playing fingers in the somatosensory cortices (9) as well as having an increased functional connectivity of insula-based networks (10). Both, the insula and the somatosensory cortices are part of the brain network responsible for pain processing (11). The high prevalence of pain syndromes, together with the aforementioned neuroplastic alterations of brain areas associated with the nociceptive system makes professional musicians an interesting group to research the influence of neuroplasticity on nociception.

For that purpose, contact heat evoked potentials (CHEPs) have been shown to be a reliable, reproducible, objective and non-invasive method to research the integrity of the nociceptive system (12–17). However, it has been shown that the baseline temperature and the heating rate of the noxious heat stimulus has a big influence on the amplitude and the latency of the CHEPs’ components (15, 18, 19). It has even been argued, that normative values for the latency and amplitude of the evoked potentials depend on the experimental setup and can thus vary considerably, despite using comparable stimulation paradigms (13, 14, 16). Thus, for reproducibility and to be able to compare studies regarding their CHEPs the stimulus used has to be appropriately quantified.

Therefore, the objectives of this study are twofold. Firstly, we present the results of a preliminary CHEPs study, where we stimulated hands and feet of musicians and non-musicians with two different stimulation intensities. We discuss the influence of the stimulation conditions, stimulation location and, most importantly, the differences between musicians and non-musicians. We hypothesize significant differences of the CHEPs regarding the different stimulation conditions, as well as significant differences regarding the latency of the CHEPs between the stimulation locations. We furthermore hypothesize differences between the groups regarding the latency of the CHEPs. Secondly, we investigate the influence of different stimulation devices on the evoked potentials by measuring the temperature curves, which are used as stimuli to evoke the respective potentials. For this we measured the temperature curves of two devices with the exact same settings: the PATHWAY system, a stimulation device commonly used in the literature (13–16, 19, 20) and our stimulation device, the TSA2. We compare the temperature curves as well as the resulting CHEPs . We hypothesize, that we can explain CHEPs differences between the literature and our measurements by the different stimulation devices.

## 2 Material and methods

### 2.1 Participants

For this preliminary study, 15 healthy musicians and 15 healthy non-musicians were recruited. The participants sex was assigned based on their self report. Each group consisted of 9 female and 6 male participants. Mainly, musicians were recruited whose primary instrument has a focus on fine motor movements of the hands, such as violinists and pianists. Based on their questionnaires, their average cumulative practice time was 7,058.86 h ± 3,215.33 h and their average daily practice time at the time of the study was 2.66 h ± 1.1 h. The control group consisted of musically naive individuals who had not played an instrument for at least 10 years. Inclusion criteria were being older than 18 years, no neurological or psychiatric conditions, no pregnancy and no pain medication at least 24 h prior to the experiment, as well as no acute or chronic pain at the time of the experiment. A more comprehensive description of the study cohort can be found elsewhere(21).

All participants provided written informed consent and all procedures described are in accordance with the declaration of Helsinki 1964 and are approved by the ethics committee of the Medical University Hannover (MHH; Reference: 10328_BO_S202).

### 2.2 Study protocol

Prior to the experiment, the participants were informed about the proceedings and asked to fill out questionnaires concerning demographics and psychometric measures related to pain, which are named in the appendix. They received five pain stimuli at the same location as was done in the experiment to familiarize themselves with the experiment. Afterwards the electrodes for the electroencephalogram (EEG) were applied. Each participant was stimulated at both hands and both feet in a random order with two different stimulation intensities. At the hands the stimuli were applied at the C6 dermatome between the thumb and the index finger, while at the feet the stimuli were applied at the L5 dermatome next to the big toe. For each condition, they received 15 stimuli. Roughly 2 s after each stimulus, participants were asked to rate the pain on a numeric rating scale between 0 and 10, 0 being no pain and 10 being the worst pain imaginable.

### 2.3 Stimulation device and recording setup

For the contact heat stimulation the TSA 2 (Medoc, Israel) was used. The 2.4 cm × 2.4 cm contact plate of the thermode delivered the stimuli with a randomized inter stimulus interval between 8 s and 12 s. The device is capable of a heating rate of upto 70 °C/s and a cooling rate of upto 40 °C/s, as reported by the manufacturer. The peak temperature for each stimulus was 52 °C with a baseline temperature of 42 °C for more painful stimuli and 35 °C for less painful stimuli, as higher baselines have been shown to result in a more painful sensation(22, 23)but result in an enhanced CHEPs signal quality(15).

For the EEG recording, the electrodes were placed according to the international 10-20 system. One electrode was placed at the Cz position to record the cortical pain response, electrodes placed at the Fp1, Fpz and Oz positions were used to record ocular and α-wave artifacts, respectively. The reference electrode was placed on the nose. All active 9 mm Ag/AgCl cup electrodes were prepared with 70% isopropanol, Abralyt 2,000 abrasive electrolyte gel (EASYCAP, GmbH), and SuperVisc HighViscosity electrolyte gel (EASYCAP, GmbH) and all impedances were kept below 10 kΩ. The EEG data was recorded with a sampling frequency of 2,500 Hz with the BrainVision Recorder software (Version 1.24.0101, BrainProducts, GmbH).

The literature values used for comparison with our CHEPs data with a stimulation at the hands were recorded by Jutzeler et al. (14) and Kramer et al. (15, 19). The stimulation device used in these studies was a PATHWAY Pain & Sensory Evaluation System (Medoc Ltd., Ramat Yishai, Israel) with a 2.7 cm × 2.7 cm contact plate CHEPs thermode. The recording conditions such as stimulation site and the EEG setup were the same, except that the aforementioned studies used an averaged reference at the ears, where as we used a reference electrode at the nose. For the stimulation protocols the exact same parameters were used as well.

### 2.4 Analysis and statistics

The EEG data was processed using a self written user interface utilizing the *python* package *mne* (version 0.24.0) (24). The data was bandpass filtered between 0.1–300 Hz and notch filtered with 50 Hz. Only if there were obvious blinking artifacts in the data of the Fp1 electrode, an independent component analysis (ICA) was used to filter out these artifacts. An ICA was used for 40 of the 240 measurements. The EEG data from the Cz electrode was partitioned into epochs 0.1 s pre-stimulus and 1 s post-stimulus, with the baseline estimated with the data within 0.1 s before the stimulus. Epochs were rejected only if there were unfixable artifacts, e.g., muscle artifacts. The remaining epochs were then averaged to obtain the contact heat evoked potentials. Of the 3,600 epochs 409 were rejected, yielding on average 13.3 out of 15 epochs per CHEP. It has previously been recommended to only use CHEPs with an amplitude larger than 10 μV (14). We acquired a CHEP with amplitudes above 10 μV for both baseline temperatures for every participant and all their extremities. No participant or measurement had to be excluded.

To investigate the influence of the different baseline temperatures (B), the stimulation location (L) and group, i.e., musician/non-musician (G) on the CHEPs parameters, a hierarchical linear mixed effects model was implemented in *R* (version 4.2.3) using the package *lme4* (version 1.1-32). The variables B, L and G are factor variables, where musicians are the baseline for the variable G, the baseline for the variable B is given by the 42 °C baseline temperature and a stimulation at the hand is the baseline for the variable L; e.g., for the $N_{2}$ latency, the intercept describes the average $N_{2}$ latency of the cortical response to 42 °C baseline stimuli applied at the musicians’ hands. The model follows the formula C∼L∗G+B∗G+(1|ID), where ID is the individual participants ID and C represents one of the following CHEPs parameters: the $N_{2}$ latency, the $P_{2}$ latency or the amplitude A. The residuals for each model were tested for normality by visually comparing their distribution to a normal distribution of the same mean and standard deviation; statistical significance was set to α=0.05.

To investigate the influence of different stimulation devices, the $N_{2}$ latency from stimuli to the hands were compared to literature values (14, 15, 19) obtained under the exact same stimulation and recording conditions, but with a different stimulation device. In addition, we had the opportunity to measure the stimuli’s temperature curves from the PATHWAY system that had been used for the acquisition of the literature values of the $N_{2}$ latency. For a quantitative comparison, the temperature curves of both devices used for the stimulation were averaged for both baseline temperatures. To that end, the devices’ temperature readouts, which are automatically recoded during the experiments, were used. The readouts include the time of the stimulus onset, which was used to align the temperature curves of the stimuli. For each time point within roughly 2 s after the onset, the temperature of all stimulus curves at that time point was averaged to compute an averaged temperature curve for each device and each condition. The heating rate of the averaged temperature curves was computed by dividing the temperature difference from baseline to peak temperature by the time between the stimulus onset and reaching the peak temperature. The devices start with no time difference, but, due to their different heating rates, they reach their peak temperature at a maximal time difference; their average time difference is therefore half of their maximal time difference. For the PATHWAY system, 46 and 70 temperature curves were used to compute the average temperature curve for the 42 °C baseline and the 35 °C baseline, respectively, resulting in one average temperature curve for each baseline temperature. For the TSA2, 1,800 temperature curves were used for each baseline temperature to compute an average temperature curve.

The temperature readouts of both devices were investigated by a self written python script.

## 3 Results

All participants completed the experiment and there was no missing data. As this paper focuses on the CHEPs data, the results for the subjective pain rating can be found elsewhere (21).

### 3.1 Influence of group, location and baseline temperature on the CHEPs parameters

**Figure 1:**
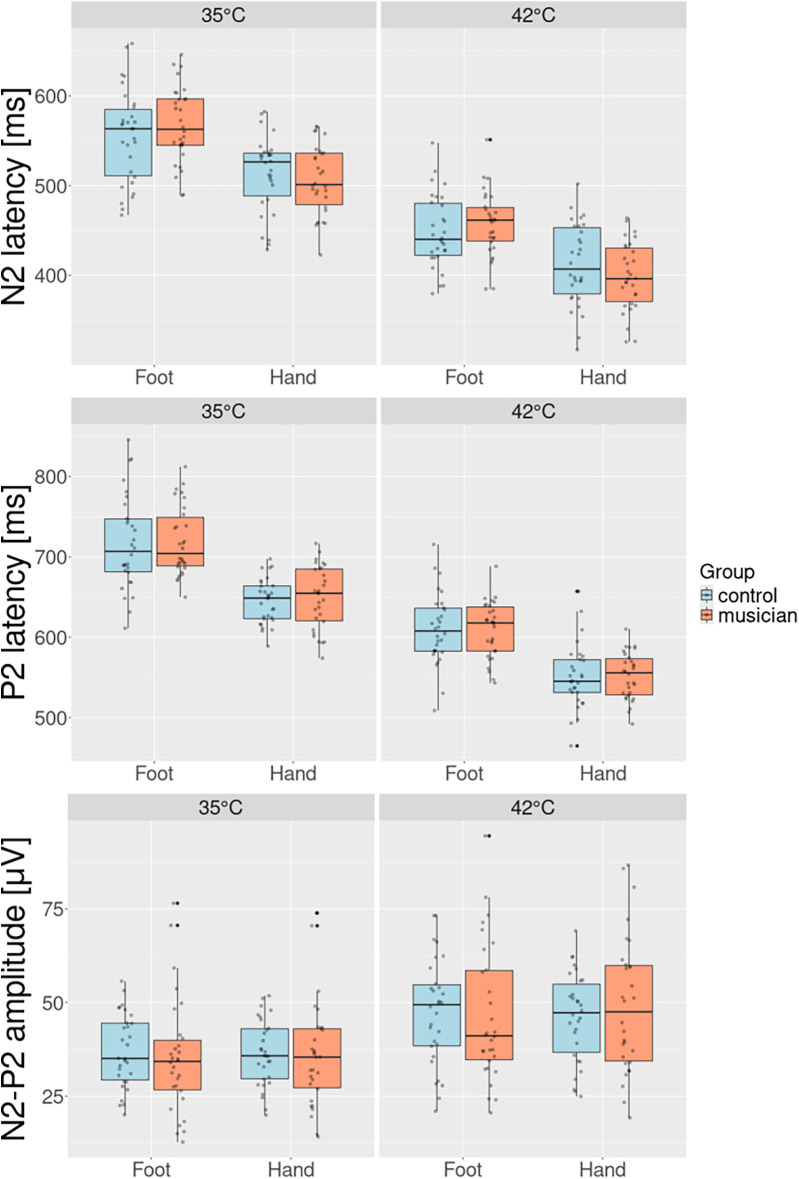
Group comparison for $N_{2}$ and $P_{2}$ latency and the $N_{2}-P_{2}$ amplitude for both stimulation sites and both baseline temperatures. The increased baseline results in a significantly shorter latency and significantly higher amplitude. Musicians have the tendency to have a shorter $N_{2}$ latency at the hands and a significantly longer $N_{2}$ latency at the feet. Musicians have a general tendency to have longer $P_{2}$ latency.

The results for the CHEPs parameters for all conditions can be seen in Table 1; they are visualized in Figure 1. The results of the linear mixed effects model are depicted in Table 2. There was no significant effect of being in a certain group on any of the CHEPs parameters. With regard to location, there were significantly longer $N_{2}$ (59.88 ms, $p<2\cdot 10^{-16})$ and $P_{2}$ (63.4 ms, $p<2\cdot 10^{-16})$ latencies at the feet compared to the hands. There was a significant difference for all CHEPs parameters between the different baseline temperatures: the $N_{2}$ latency was 108.75 ms longer $\left(p<2\cdot 10^{-16}\right)$, the $P_{2}$ latency was 104 ms longer $\left(p<2\cdot 10^{-16}\right)$ and the amplitude A was 12.41 μV lower $\left(p=4.61\cdot 10^{-16}\right)$ for the 35 °C baseline as compared to the 42 °C baseline protocol. There was a significant group difference with the interaction with the location, where musicians had a significantly larger $N_{2}$ latency difference between hands and feet of 20.86 ms (p=0.0045) compared to the non-musicians.

**Table 1:** Average values for the $N_{2}$ and $P_{2}$ latency and the amplitude A of the CHEPs for both baseline temperatures and both stimulation locations.

		35 °C		42 °C	
		Foot	Hand	Foot	Hand
$N_{2}$	Control	555.27	513.36	448.49	412.35
	Musician	567.4	506.57	457.71	398.76
$P_{2}$	Control	717.13	645.05	610.21	550.83
	Musician	719.17	649.77	609.17	551.77
A	Control	37.05	36.47	47.46	46.12
	Musician	35.24	35.77	46.63	49.19

The unit of the $N_{2}$ and $P_{2}$ latency is [ms] and the unit of the amplitude A is [μV].

**Table 2:** Estimated effects of group G, stimulation location L, baseline temperature B and the interactions G:L and G:B on the $N_{2}$ and $P_{2}$ latency and the amplitude A.

	G	L	B	G:L	G:B
$N_{2}$	12.61	59.88	108.75	−20.86	−4.857
p	0.346	$<2\cdot 10^{-16}$	$<2\cdot 10^{-16}$	0.0045	0.504
$P_{2}$	−1.12	63.4	104	2.333	−3.427
p	0.927	$<2\cdot 10^{-16}$	$<2\cdot 10^{-16}$	0.768	0.664
A	−2.370	−1.543	−12.407	2.503	2.377
p	0.621	0.273	$4.61\cdot 10^{-16}$	0.209	0.233

Group has no significant effect. The interaction G:L has a significant effect on $N_{2}$, suggesting that musicians have significantly larger $N_{2}$ latency difference between hands and feet. $N_{2}$ and $P_{2}$ latency are significantly longer for stimulation at the feet. $N_{2}$ and $P_{2}$ latency are significantly longer for a lower baseline temperature. The amplitude A is significantly lower for a lower baseline temperature. The unit of the estimates for $N_{2}$ and $P_{2}$ is [ms] and the unit for the amplitude A is [μV]. For each model there where 240 observations across the 30 participants. For each of the models for $N_{2}/P_{2}/A$ the variance of the residuals $\sigma^{2}$, the variance of the intercept $\tau_{00ID}$, the interclass correlation ICC and the marginal $R^{2}$ as well as the conditional $R^{2}$ are as follows: $\sigma^{2}=789.55/932.1/59.24$, $\tau_{00ID}=1,014.7/746.1/146.55$, ICC=0.56/0.44/0.71, $R_{\text{marginal}}^{2}=0.659/0.687/0.136$, $R_{\text{conditional}}^{2}=0.851/0.826/0.751$.

Bold values indicate *p* values below 0.05.

### 3.2 Comparison between two stimulation devices and comparison to literature

Table 3 shows the comparison of the $N_{2}$ latency measured with a stimulation at the C6 dermatome, for both baseline temperatures between the study discussed in this report and literature values measured by Jutzeler et al. (14) and Kramer et al. (15, 19). The table shows that we measure a systematically longer $N_{2}$ latency. For the 42 °C baseline the $N_{2}$ latency of the potentials resulting from a stimulation with the TSA2 is 115.2 ms longer than the $N_{2}$ latency recorded with the PATHWAY system. For the 35 °C baseline the $N_{2}$ latency recorded with the TSA2 is 92.98 ms longer than the one recoded with the PATHWAY system. On average, this is a discrepancy of 104.09 ms, despite otherwise identical recording conditions.

**Table 3:** Comparison of the $N_{2}$ latency for stimulation at the hands with literature values.

Hands				
42 °C				
$N_{2}$	$N_{2}^{1}$	$N_{2}^{2}$	$N_{2}^{3}$	
405.6±41.9	287±22.8	308.71±27.8	275.5±23.3	
$N_{2}-N_{2}^{i}$	118.6	96.89	130.1	∅115.2
35 °C				
$N_{2}$	$N_{2}^{1}$	$N_{2}^{2}$	$N_{2}^{3}$	
510±40.1	381.1±31.9	432.9±104.03	437.07±102.33	
$N_{2}-N_{2}^{i}$	128.9	77.1	72.93	∅92.98

All given values are in [ms]. For both baseline temperatures the measured $N_{2}$ values are considerably larger than the literature values. The values for $N_{2}^{1}$, $N_{2}^{2}$ and $N_{2}^{3}$ are taken from the following sources respectively: Jutzeler et al. (14), Kramer et al. (15) and Kramer et al. (19).

**Figure 2:**
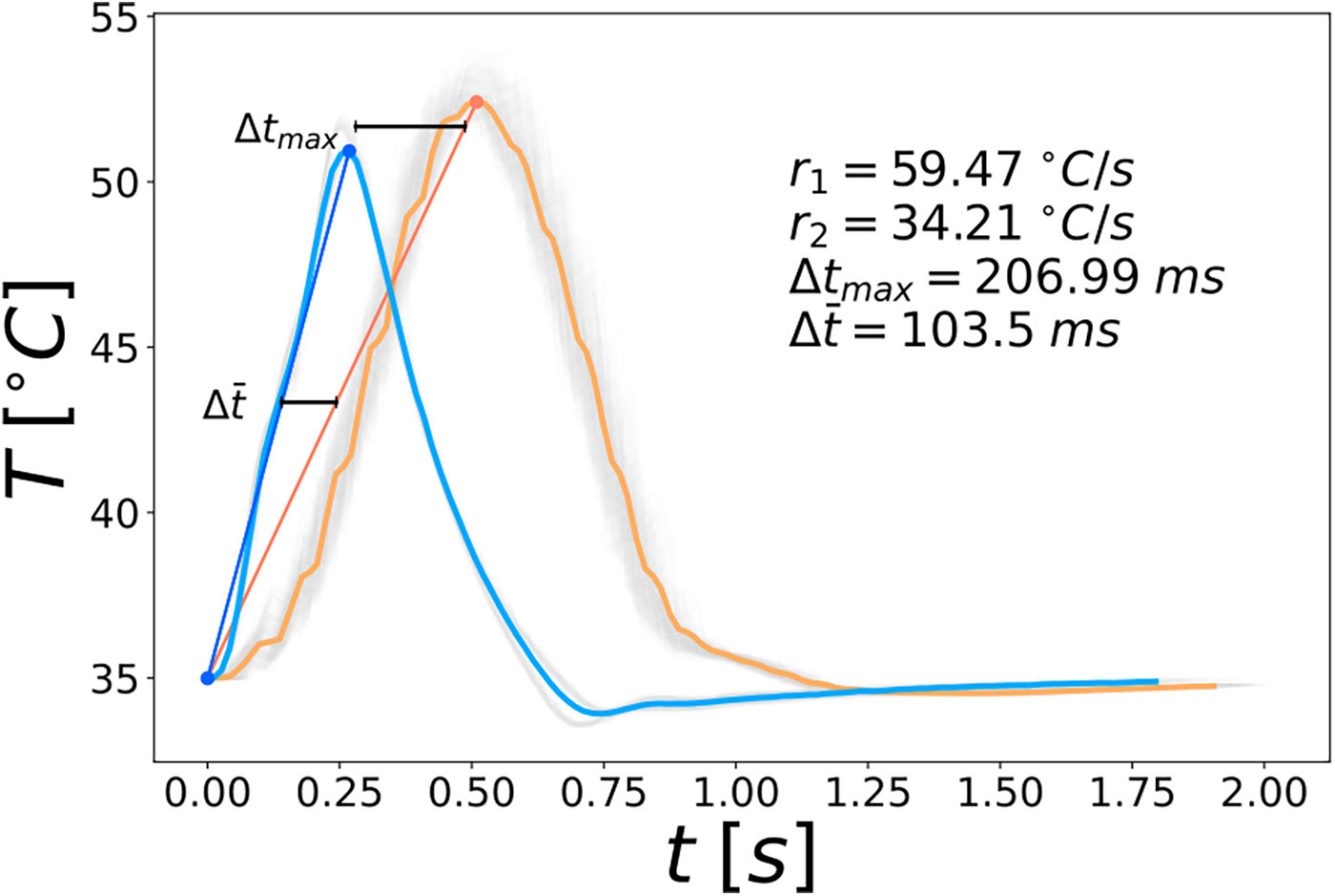
Averaged temperature curves with their respective heating rates and the average time difference for the 35 °C baseline. The temperature curves of the single stimuli are shown in the background in gray. The stimulation devices are color coded; blue: PATHWAY system, orange: TSA2 system.

As an example for the quantitative comparison of the stimulation devices, Figure 2 shows the grand averaged temperature curves for the PATHWAY system (blue), used in the literature and the TSA2 system (orange) that was used in this study. Depicted are the temperature curves that start at the 35 °C baseline and upon reaching their peak temperature, return to this baseline. Despite using identical parameters in the software used to control the stimulation devices (15 stimuli, baseline temperature: 35 °C, target temperature: 52 °C, fastest possible cooling/heating, random ISI between 8-12 s), the temperature curves look considerably different. The TSA2 has a longer wind up until the temperature increases. Therefore, it reaches it’s maximal temperature considerably later than the PATHWAY system. Additionally, it has a slower cooling compared to the PATHWAY system. Quantitatively, for the 35 °C baseline stimulus, the TSA2 reaches its maximal temperature after 0.487 s with a heating rate of 34.21 °C/s, while the PATHWAY system reaches its maximal temperature after 0.280 s and has a heating rate of 59.47 °C/s. The devices reach their peak temperature with a time difference of 206.99 ms, which yields an average time difference of 103.5 ms. For the 42 °C baseline, the TSA2 reaches its maximal temperature after 0.386 s with a heating rate of 25 °C/s and the PATHWAY reaches its maximal temperature after 0.174 s with a heating rate of 55.48 °C/s, which leads to a maximal time difference of 212.09 ms and an average time difference of 106.05 ms. In conclusion, the different devices operate with an average time difference of 104.78 ms.

## 4 Discussion

In the present study we investigated the differences in CHEPs parameters between musicians and non-musicians. We found a larger $N_{2}$ latency difference between hands and feet for the musicians, compared to the non-musicians. Additionally, we quantitatively investigated the influence of stimulation devices on the CHEPs data. We found that the latency difference between potentials induced by different stimulation devices can be explained quantitatively by the different heating rates of the devices.

### 4.1 Contact heat evoked potentials

**Figure 3:**
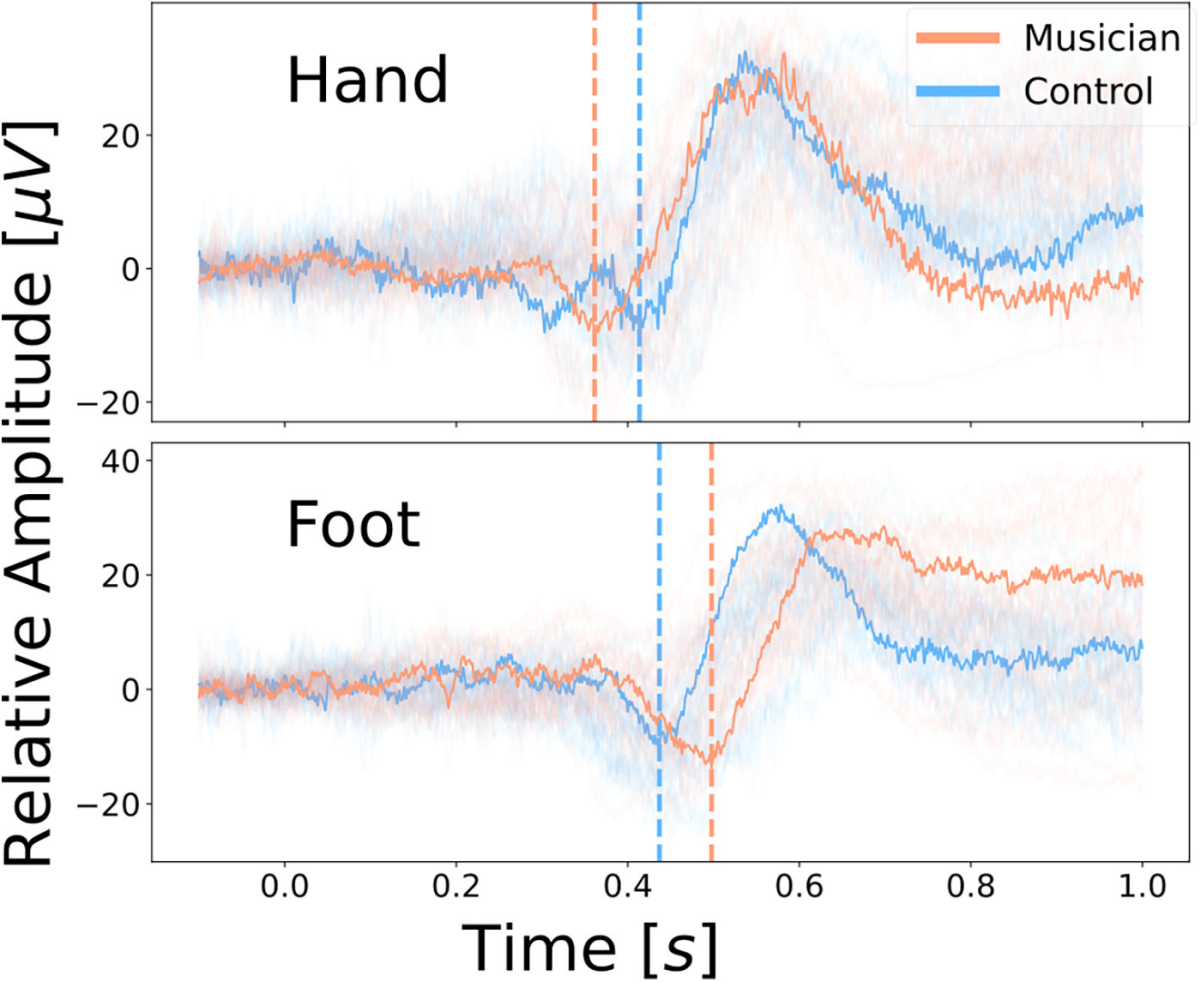
Relative amplitude of the cortical response to painful heat stimuli with a baseline temperature of 42 °C at the hand (top) and the foot (bottom) with respect to time. The highlighted examples visualize musicians (orange) having a larger latency difference between hands and feet compared to the controls (blue).

Our participants showed a significantly longer $N_{2}$ latency for the feet compared to the hands, in agreement with multiple other studies (13, 14, 16). This result is not surprising, since the distance between the location of stimulation and the cortex is longer for the feet than for the hands. Interestingly, the estimate of the interaction between the group and the location of stimulation shows that this tendency is significantly more pronounced for musicians than for non-musicians. This means that the latency difference between the hands and the feet is larger for musicians than for non-musicians, indicating that musicians have a shorter $N_{2}$ latency at their hands and a longer $N_{2}$ latency at their feet. This result can be seen from Table 1; additionally, Figure 3 visualizes the larger latency difference between hands and feet of musicians compared to non-musicians. Figure 3 shows the relative amplitude of the cortical response to painful stimuli at the hand (top) and the foot (bottom) with respect to time. The amplitude is scaled by the smallest amplitude of all potentials to make it easier to compare the potentials visually. The highlighted examples exaggeratedly visualize the shorter latency at the hand of the musician (orange) and the longer latency at their foot compared to the non-musician (blue).

We suspect that the $N_{2}$ latency alteration in musicians indicates a neuroplastic adaptation of the nociceptive system, which can indeed be induced by musical training (9, 10, 25, 26). Likely due to their higher sensitivity to pain and to mechanical stimuli (9, 27), musicians experience pain more often during their career, as is evidenced by their high prevalence of pain syndromes (2–4). At the same time, we argue that professional musicians often endogenously inhibit their pain, for example to keep practicing despite having pain to be able to compete in their stressful and highly competitive environment (28). Enhanced pain inhibition in musicians has likewise been anticipated by Zamorano et al. (26). The endogenous pain inhibition is a top-down controlled aspect of the nociceptive system that can be engaged intentionally or unintentionally (29–31). It is part of the central nervous system and works via descending pathways that inhibit nociceptive signaling at the level of the first synapse (30, 32, 33). Thus, we conclude that during their training of more than 10,000 h (34) musicians have more cumulative activity in both their ascending nociceptive pathways as well as their descending anti-nociceptive pathways compared to non-musicians. As enhanced neuronal activity has been shown to be associated with an increased myelination (6–8), the nerve fibres of the aforementioned pathways might be more myelinated in musicians than in non-musicians leading to an increased conduction velocity along said fibres. The altered $N_{2}$ latency in musicians might thus be explained by an interplay of more myelinated ascending and descending nerve fibres. Namely, the shortened $N_{2}$ latency at the hands of musicians could be explained by the enhanced conduction velocity of the ascending nerve fibres being the dominating factor, while the elongated $N_{2}$ latency at the feet might conversely be explained by the enhanced conduction velocity of the descending inhibitory fibres being the dominating factor. The latter can be thought of as a retardation of the afferent volley due to a fast top-down pain inhibition. The reason for the difference in which factor plays the dominant role in the hands compared to the feet might plausibly be that for the feet a larger proportion of the distance in the nociceptive system between the location of stimulation and the cortex is governed by the central nervous system. Since pain inhibition is a central phenomenon, it might therefore be more relevant for the $N_{2}$ latency at the feet as compared to the hands. Note, that a more frequent engagement of the endogenous pain inhibition in musicians does not necessarily contradict their aforementioned enhanced pain sensitivity; it merely implies that they inhibit pain more often, it does not mean that they feel less pain. However, we acknowledge that this explanation is speculative and more research regarding musician’s pain transmission and inhibition is needed to replicate and explain this surprising result.

In our study population, we found no significant group effects or group interaction effects on the $P_{2}$ latency or the amplitude of the contact heat evoked potentials measured at the Cz electrode. In a publication about the subjective pain ratings during this preliminary study, we found that musicians showed significantly higher pain ratings as compared to non-musicians (21). Since, at least partially, the amplitude and the $P_{2}$ component are associated with higher cortical functions such as evaluation (35, 36), one might expect a larger amplitude or an alteration of the $P_{2}$ component as well. However, this aspect of subjective evaluation is more associated with the prefrontal areas of the brain (35, 36). Given that we recorded the CHEPs at the Cz location, which is close to the motor areas of the brain, the group differences in subjective evaluation might not sufficiently be captured by our reduced EEG setup. Future studies should focus on linking the group differences regarding subjective pain ratings to group differences regarding the amplitude and the $P_{2}$ component of evoked potentials in the prefrontal areas.

Additionally, the models show a significant effect of the different baseline protocols on all three of the CHEPs parameters. The effect of an increased baseline stimulation resulting in a decreased $N_{2}$ and $P_{2}$ latency and an increased amplitude A, has previously been shown (14, 16). The increased latency for the decreased baseline is to be expected, because it takes longer for the stimulus to reach the maximal temperature, which means that it lasts longer. This has additionally been shown by Kramer et al. (15). The longer duration of the stimulus also explains the decreased amplitude for the lower baseline, as it leads to a less synchronized afferent volley, resulting in a decreased amplitude (18).

### 4.2 Stimulation device

In this report, we focused on the comparison of the $N_{2}$ latency for stimuli at the hands between two different stimulation devices. We chose the $N_{2}$ for comparison for two reasons: firstly, because the $N_{2}$ directly comprises temporal information of when the cortical response is measured in relation to the stimulus and secondly, because it is a very pronounced immediate component of the evoked potential. Moreover, we wanted to compare to the aforementioned literature values (14, 15, 19).

In previous studies, it has been shown that e.g., age (14), stimulation location (16) or a different laboratory (13) influence the CHEPs parameters. With the same settings for the stimulation device as in the literature, we would have expected similar CHEPs. However, despite the exact same settings, the two different stimulation devices produced considerably different temperature curves. We showed that the time difference of 104.09 ms between our measured $N_{2}$ latency and the $N_{2}$ latency from the literature can be explained by the average time difference in the temperature curves between both devices of 104.78 ms. This means that the different heating rates of the stimulation devices explain the different $N_{2}$ latency very well, underlining the importance of comparable temperature curves to measure comparable evoked potentials. The influence of different heating rates on CHEPs has already been shown in other studies (18). However, the significance of this report is firstly, to show that the same settings of the stimulation devices still can result in different stimuli and secondly, to quantitatively show the significant influence different temperature curves have on the resulting evoked potentials. Therefore, providing more information about the actual temperature curves in addition to the software settings is important for conclusive results regarding the integrity of the nociceptive system using CHEPs. We recommend to measure and evaluate the temperature curves used during the experiment and to report at least the measured heating rate and the time for reaching the maximal temperature after the onset. Depending on the experiment ,other quantities, like the measured total duration of the stimulus might be important as well.

### 4.3 Limitations

The results of this study need to be viewed in the context of the study’s limitations. For a deeper and more quantitative understanding of how the temperature curve influences the contact heat evoked potential, multiple stimulation devices with multiple different sets of settings should be tested. Regarding the group comparison, the unbalanced sex distribution (18 females, 12 males) in our preliminary study population might skew the results of the CHEPs. Furthermore, the small sample size of 30 participants might limit the generalizability of the results. A larger study population with a balanced sex distribution is needed to reproduce and contextualize the results. Given the reduced EEG setup of only four electrodes, spacial aspects of the cortical response can not sufficiently be resolved. A setup with 64 electrodes would overcome this limitation. Due to the special relationship musicians have towards pain, it has to be noted that qualitative data from interviews or questionnaires should be included to enrich the interpretation of the results.

## 5 Conclusion

We showed that musicians, indeed, show differences in their nociception. It is worthwhile to investigate these differences further, for instance by looking at other latency components as the $N_{1}$ latency or the duration of the cortical response. Understanding pain in musicians might not only enable us to find better and specific treatments, but might also shed light on how neuroplasticity can influence nociception in general. In particular, it would be very insightful how the musicians enhanced sensitivity and their hypothesized altered inhibition manifests differently at the hands compared to the feet. For that purpose, contact heat evoked potentials present a reliable, objective and non-invasive method to investigate the integrity of the nociceptive system (12–16). But to ensure comparability of the evoked potentials, the temperature curves of the stimulation devices must be comparable as well. As we have shown, despite the exact same settings in the stimulation devices, the temperature curves can differ considerably, leading to different evoked potentials. This has big implications regarding the comparability of study results, as large differences might be induced by the stimulation device. We suggest to report more information on the temperature curves used in CHEPs; e.g., the measured heating rate and the actual baseline and peak temperature, as well as the time between onset and the maximal temperature and the total duration of the stimulus. This will further facilitate comparable research on pain in groups of people frequently affected by it, such as musicians. This, in turn, will help us to further understand the vastly complex topic of pain to hopefully mend its consequences.

## Data Availability

The raw data supporting the conclusions of this article will be made available by the authors, without undue reservation.

## Appendix

### Questionnaires

As the results of the questionnaires are not part of this study, they are only mentioned in the appendix for completeness. The following questionnaires were used: the German version of the Pain Catastrophizing Scale (PCS), the trait section of the Wettkampf-Angst-Inventar (WAI-T, the Competition Anxiety Inventory), the German version of the revised Pain Attitudes Questionnaire (PAQ-R) and the trait section of the State-Trait Anxiety and Depression Inventory (STADI).
